# Excitation spectra in fluids: How to analyze them properly

**DOI:** 10.1038/s41598-019-46979-y

**Published:** 2019-07-19

**Authors:** Nikita P. Kryuchkov, Lukiya A. Mistryukova, Vadim V. Brazhkin, Stanislav O. Yurchenko

**Affiliations:** 1 Bauman Moscow State Technical University, 2nd Baumanskaya street 5, Moscow, 105005 Russia; 2 Institute for High Pressure Physics RAS, Kaluzhskoe shosse, 14, Troitsk, Moscow, 108840 Russia

**Keywords:** Chemical physics, Fluids

## Abstract

Although the understanding of excitation spectra in fluids is of great importance, it is still unclear how different methods of spectral analysis agree with each other and which of them is suitable in a wide range of parameters. Here, we show that the problem can be solved using a two-oscillator model to analyze total velocity current spectra, while other considered methods, including analysis of the spectral maxima and single mode analysis, yield rough results and become unsuitable at high temperatures and wavenumbers. To prove this, we perform molecular dynamics (MD) simulations and calculate excitation spectra in Lennard-Jones and inverse-power-law fluids at different temperatures, both in 3D and 2D cases. Then, we analyze relations between thermodynamic and dynamic features of fluids at (Frenkel) crossover from a liquid- to gas-like state and find that they agree with each other in the 3D case and strongly disagree in 2D systems due to enhanced anharmonicity effects. The results provide a significant advance in methods for detail analysis of collective fluid dynamics spanning fields from soft condensed matter to strongly coupled plasmas.

## Introduction

Knowledge of collective excitation spectra in condensed matter opens a way for proper interpretation and understanding of most phenomena and related properties, including elastic, thermodynamic, and transport ones^1–3^. Apart from condensed matter, excitation spectra provide a useful tool for the analysis of phenomena in strongly-coupled plasmas. For this reason, the calculation of excitation spectra in fluids is a problem of high significance and broad relevance to condensed matter, chemical physics, physical chemistry, physics of plasmas, materials science, and soft matter.

In crystals under conditions far from melting, harmonic theory of crystal lattice can typically be applied, since (e.g., at low temperature and/or high pressure) effects of anharmonicity are weak and collective excitations can be treated as a set of noninteracting harmonic plane waves, referred to as phonons^2^. With increasing temperature, the (typically weak) effects of anharmonicity lead to interactions between phonons. These interactions result in a finite lifetime and frequency shift of the phonons, which can be analyzed using perturbation theory^2^, except for strongly-anharmonic crystals^4–7^.

In contrast to crystals, the excitation spectra in liquids are less understood, since there is no small parameter related to anharmonicity^8^. Effects of interplay between oscillating and damped collective behaviors in fluids are often treated in the framework of generalized hydrodynamics^9–16^ or using the quasi-localized crystalline approximation (QLCA) and its modifications^17–31^. However, both these approaches have fundamental disadvantages, since anharmonicity effects are taken into account phenomenologically. Recently, excitation spectra in fluids have become of great interest in context of studies concerning the thermodynamics and collective dynamics of fluids^8,26,32–62^, and, in particular, crossover from fluid- to gas-like collective dynamics (Frenkel line)^8,32,34–41,47,48,50–53,56,58^ and in the framework of inelastic X-ray or neutron scattering experiments^63–76^. Note that these experiments have already revealed a problem of mixing of longitudinal and transverse modes, which plays an important role in a proper analysis of excitation spectra as will be shown below. Dynamic and thermodynamic features attributed to the Frenkel line are typically considered using different methods to obtain excitation spectra of fluids from MD simulation results. However, it is still unclear which method for the calculation of excitation spectra (frequencies and damping rates) in fluids is the most suitable and provides the most accurate and consistent results, as well as how different widely-used approaches to this important problem are consisted with each other.

In this work, we analyze different methods for the calculation of excitation spectra in fluids, including the analysis of velocity current spectral maxima, its longitudinal and transverse components, and a method involving the two-oscillator model for the analysis of total velocity current spectra. We find that the most accurate and consistent results are provided by the two-oscillator model, while other approaches can be used only for rough estimation of the low-frequency branch of dispersion relations at low temperatures, but become unsuitable even for this aim at high temperatures and wavenumbers. To confirm this conclusion, we analyze excitation spectra in fluids, using MD simulations of Lennard-Jones fluids and fluids of particles interacting with each other via inverse-power-law repulsive potentials (∝1/*r*^12^ and 1/*r*^8^) at different temperatures. Then, we analyze thermodynamic, collective, and individual dynamic features associated with crossover from liquid- to gas-like dynamics of fluids (Frenkel line) in 3D and 2D fluids. In conclusion, we discuss how longitudinal and transverse current spectra are changed at the transition from collective to individual particle dynamic regime, corresponding to excitations with high wavenumbers.

## Results and Discussion

### Separate mode analysis versus the two-oscillator model

To compare different approaches to the calculation of excitation spectra in fluids, we performed MD simulations whose details are described in Methods. We considered several model systems at the same density and different temperatures, including a Lennard-Jones fluid, and fluids of particles interacting via inverse power law repulsion ∝1/*r*^12^ (IPL12), and a more soft variant ∝1/*r*^8^ (IPL8), to analyze features arising in systems with different kinds of interactions. We used the Lennard-Jones system as a representative and well-studied model describing noble gases^5,36,39,77^, while the inverse-power-law fluids^5–7,21,26^ were considered to compare the results with the Lennard-Jones system and, hence, to reveal features associated with attraction and repulsion softness.

For the systems under consideration, after particle velocities were obtained from MD simulations, we calculated the velocity current spectra^9^

1 $${C}_{L,T}({\bf{q}},\omega )=\int \,dt\,{e}^{i\omega t}{\rm{Re}}\,\langle {{\bf{j}}}_{L,T}({\bf{q}},t){{\bf{j}}}_{L,T}(\,-\,{\bf{q}},0)\rangle ,$$

where ${\bf{j}}({\bf{q}},t)={N}^{-1}\,{\sum }_{s}\,{{\bf{v}}}_{s}(t)\,\exp (i{\bf{q}}{{\bf{r}}}_{s}(t))$ is the velocity current; ${{\bf{v}}}_{s}(t)={\dot{{\bf{r}}}}_{s}(t)$ is the velocity of the *s*-th particle; the summation is performed over all *N* particles in the system; ${{\bf{j}}}_{L}={\bf{q}}({\bf{j}}\cdot {\bf{q}})/{q}^{2}$ and ${{\bf{j}}}_{T}=({\bf{j}}\cdot {{\bf{e}}}_{\perp }){{\bf{e}}}_{\perp }$ are the longitudinal and transverse components of the current, respectively; ${{\bf{e}}}_{\perp }$ is a unit vector normal to **q**; the brackets $\langle \ldots \rangle$ denote the canonical ensemble average.

Since simple fluids are isotropic, velocity current spectra depend only on the frequency $\omega$ and wavenumber $q=|{\bf{q}}|$. Therefore, we can average ${C}_{L,T}({\bf{q}},\omega )$ over all directions of the wavevector **q** to suppress noise caused by a limited number *N* and finite simulation time,

2 $${C}_{L,T}(q,\omega )=\frac{1}{{N}_{q}}\,\sum _{|{\bf{q}}|=q}\,{C}_{L,T}({\bf{q}},\omega ),$$

where *N*_*q*_ is the number of directions used for averaging. Note that this approach is not suitable at $q\lesssim 2\pi$/*L*, where *L* is the size of the simulation box.

To obtain excitation spectra using ${C}_{L,T}(q,\omega )$, we assumed that the velocity current spectra corresponding to *damped oscillating excitations* are

3 $${\rm{Re}}\,\langle {{\bf{j}}}_{L,T}({\bf{q}},t){{\bf{j}}}_{L,T}(\,-\,{\bf{q}},0)\rangle \propto {e}^{-{{\rm{\Gamma }}}_{L,T}(q)|t|}\,\cos ({\omega }_{L,T}(q)t),$$

where ${\omega }_{L,T}$ and ${{\rm{\Gamma }}}_{L,T}$ are the frequencies and damping rates of longitudinal and transverse modes, respectively. Using Eq. (3), we obtain the double-Lorentzian form of velocity current spectra

4 $${C}_{L,T}(q,\omega )\propto \frac{{{\rm{\Gamma }}}_{L,T}(q)}{{(\omega -{\omega }_{L,T}(q))}^{2}+{{\rm{\Gamma }}}_{L,T}^{2}(q)}+\frac{{{\rm{\Gamma }}}_{L,T}(q)}{{(\omega +{\omega }_{L,T}(q))}^{2}+{{\rm{\Gamma }}}_{L,T}^{2}(q)}.$$

Fitting (separately) velocity current spectra (2) (obtained from MD) by the Lorentzian fit (4), we calculated the frequencies and damping rates of longitudinal and transverse excitations (*single mode analysis*).

Another approach to the same problem (which is, however, rarely used) is related to the joint mode analysis (*two-oscillator model*), at which the total velocity current spectrum $C(q,\omega )={C}_{L}(q,\omega )+(D-1){C}_{T}(q,\omega )$ is fitted by the sum of high- and low-frequency double-Lorentzian terms

5 $$\begin{array}{lll}C(q,\omega ) & \propto  & \frac{{{\rm{\Gamma }}}_{L}}{{(\omega -{\omega }_{L})}^{2}+{{\rm{\Gamma }}}_{L}^{2}}+\frac{{{\rm{\Gamma }}}_{L}}{{(\omega +{\omega }_{L})}^{2}+{{\rm{\Gamma }}}_{L}^{2}}\\  &  & +\,\frac{(D-1){{\rm{\Gamma }}}_{T}}{{(\omega -{\omega }_{T})}^{2}+{{\rm{\Gamma }}}_{T}^{2}}+\frac{(D-1){{\rm{\Gamma }}}_{T}}{{(\omega +{\omega }_{T})}^{2}+{{\rm{\Gamma }}}_{T}^{2}},\end{array}$$

where *D* is the space dimension.

**Figure 1:**
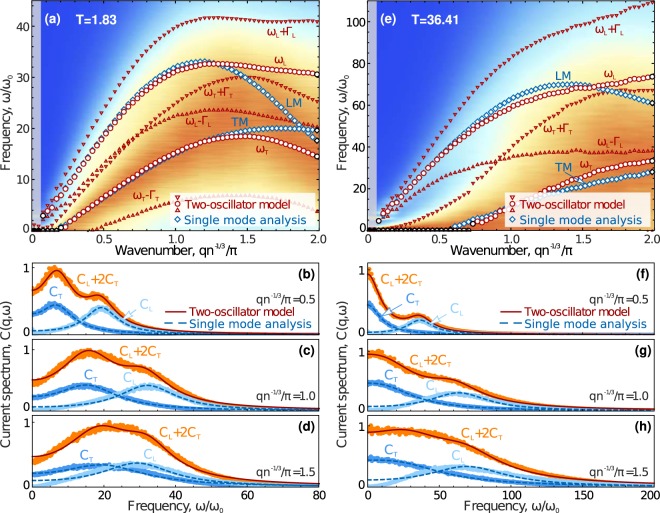
Velocity currents and excitation spectra in a 3D Lennard-Jones fluid. (**a**) Velocity current spectrum $C(q,\omega )$ shown in color-coded format (normalized to maximum for each *q*) at $T=1.83$ and $n=1$, dispersion relations ${\omega }_{L,T}(q)$ obtained using single mode analysis (blue rhombs corresponding to longitudinal (LM) and transverse (TM) modes) and the two-oscillator model (red circles), while triangles correspond to ${\omega }_{L,T}\pm {{\rm{\Gamma }}}_{L,T}$. The grey zone corresponds to the region of $q{n}^{-1/3} < 2\pi /L$ (*L* is the size of considered system). Panels (b–d) demonstrate sections of the velocity current spectrum at $q{n}^{-1/3}/\pi =0.5$, 1.0, and 1.5 obtained from MD simulations (symbols) and their fits using the two-oscillator model (5) and single mode analysis with Eq. (4), shown by the solid red and dashed blue lines, respectively. (**e**–**h**) Results obtained at $T=36.41$ and $n=1$, description is the same as in panels (a–d), respectively.

In the both methods, using single mode analysis and two-oscillator model, we obtained dispersion relations ${\omega }_{L,T}(q)$ and damping rates ${{\rm{\Gamma }}}_{L,T}(q)$. To compare the methods, we present in Fig. 1 our results for the Lennard-Jones fluid that were calculated at temperatures (a)–(d) 1.83 and (e)–(h) 36.41 (in Lennard-Jones units).

Figure 1(a) presents the total velocity current spectra $C(q,\omega )$ in a color-coded format (normalized to the maximum at each *q*). Blue rhombs correspond to the dispersion relations ${\omega }_{L,T}(q)$ calculated using single mode analysis of longitudinal (LM) and transverse (TM) modes. Results obtained using the two-oscillator model are shown by red circles for ${\omega }_{L,T}(q)$ and by triangles for ${\omega }_{L,T}(q)\pm {{\rm{\Gamma }}}_{L,T}$ to illustrate a relation between damping rates and oscillation frequencies. The damping rates obtained from single mode analysis are almost the same as those obtained using the two-oscillator model and, thus, we do not show them in Fig. 1(a,e). Numerically-obtained profiles of $C(q,\omega )$, as well as their longitudinal and transverse components ${C}_{L,T}(q,\omega )$, at different wavenumbers are shown in Fig. 1(b–d) by symbols, while the solid lines are fits by Eqs. (4) and (5). The description of the results shown in Fig. 1(e–h) is the same as in panels (a)–(d).

It is noteworthy that the both approaches yield close results, if the total current spectrum $C(q,\omega )$ has two pronounced maxima (at lower and higher frequencies, which are typically associated with longitudinal and transverse excitations, respectively). This corresponds to lower temperatures and wavenumbers corresponding to the first Brillouin pseudo-zone. Discrepancy between dispersion relations obtained using different methods increases with the temperature, as seen in Fig. 1(a,e). One can see in Fig. 1(b–d,f–h) that the current spectra obtained from MD simulations are described well by theoretical fits (4) and (5). The worst results are observed for excitations at high temperatures and short wavelengths, as indicated in Fig. 1(h). This is related to change in the shape of velocity current spectra in the high-*q* limit (see corresponding discussion below). Results of analysis using maxima of ${C}_{T}(q,\omega )$ is presented in the next section.

Note that the single mode analysis becomes unsuitable if longitudinal and transverse modes cross, as can be seen at $q{n}^{-1/3}/\pi \simeq 1.9$ in Fig. 1(a). This is because structural disorder in a fluid mixes longitudinal and transverse modes^66,67^, leading to an effective interaction between the modes and resulting in their *anticrossing* accompanied by the strong redistribution and hybridization of spectra^78^. Strictly speaking, due to the mode anticrossing, high- and low-frequency hybridized modes with mixed polarizations appear instead of longitudinal and transverse modes. Mode anticrossing should be properly taken into account in the *q*-region where the modes cross each other, and the detailed analysis of this problem stands beyond the scope of this work. However, we should stress that the mode mixing cannot be taken into account properly using single mode analysis in principle, comparing to the two-oscillator model.

### *q*-gap, overdamped, and nonoverdamped excitations

At small *q*-values, the low-frequency branch of excitations can have zero oscillation frequency ${\omega }_{T}=0$, that means the complete absence of oscillating excitations in thermodynamic equilibrium. The corresponding wavenumber range forms a gap in the *q*-space, which is referred to as the *q*-gap and is already clearly seen in Fig. 1(a,e). The long-standing problem of the calculation of the *q*-gap has recently attracted considerable interest in view of crossover between liquid- and gas-like collective dynamics in fluids with change in temperature and pressure^8,26,32,34–45,47–58^. Here, we present the calculated excitation spectra near the *q*-gap and compare the dispersion relations obtained using the two-oscillator model to the results based on the analysis of transverse current spectra.

Analysis of ${C}_{L,T}(q,\omega )$ maxima at given *q*-values is widely used to obtain dispersion relations ${\omega }_{L,T}(q)$ in crystals (see, e.g., refs ^8,32–36,38–41,52,53,58^). Such an approach is suitable in this case, since the current spectra *C*_*L*,*T*_ in crystals typically consist of narrow bands on the $(q,\omega )$ plane, while the damping rates are negligible and, thus, are assumed to be equal to zero. On the contrary, the high- and low-frequency branches of the current spectra ${C}_{L,T}(q,\omega )$ in fluids^21,26,66,67^ or crystals with developed anharmonicity^7^ are significantly broaden and overlapped with each other. For this physical reason, we should use the two-oscillator model to calculate excitation spectra, since other approaches become unsuitable.

As seen, the two-oscillator model enables in-depth analysis of excitations spectra. For instance, using model (4) for the transverse part of velocity current spectra, we readily derive the frequency ${\omega }_{m}$ corresponding to the maximum of ${C}_{T}(q,\omega )$ at a given *q* value (the dispersion relation based on the analysis of ${C}_{T}(q,\omega )$ maxima)

6 $${\omega }_{m}={\omega }_{T}\,{\rm{Re}}\,{[2\sqrt{1+{(\frac{{{\rm{\Gamma }}}_{T}}{{\omega }_{T}})}^{2}}-{(\frac{{{\rm{\Gamma }}}_{T}}{{\omega }_{T}})}^{2}-1]}^{1/2}.$$

It follows from Eq. (6) that, with an increase in ${{\rm{\Gamma }}}_{T}$/${\omega }_{T}$, the ratio ${\omega }_{m}$/${\omega }_{T}$ decreases to zero at ${{\rm{\Gamma }}}_{T}$/${\omega }_{T}=\sqrt{3}$, even at ${\omega }_{T}\ne 0$.

Near ${{\rm{\Gamma }}}_{T}$/${\omega }_{T}=\sqrt{3}$, where overdamped excitations become non-overdamped, one can expand ${\omega }_{m}^{2}$ in a *q*-series and obtain

7 $$\begin{array}{rcl}{\omega }_{m} & = & {\rm{Re}}\sqrt{{\alpha }_{\ast }(q-{q}_{\ast })+{\beta }_{\ast }{(q-{q}_{\ast })}^{2}},\\ \alpha  & = & 3(\frac{\partial {\omega }_{T}}{\partial q}-\frac{\sqrt{3}}{3}\frac{\partial {{\rm{\Gamma }}}_{T}}{\partial q}){\omega }_{T},\\ \beta  & = & \frac{3}{8}{(\frac{\partial {\omega }_{T}}{\partial q})}^{2}+\frac{3\sqrt{3}}{4}\frac{\partial {{\rm{\Gamma }}}_{T}}{\partial q}\frac{\partial {\omega }_{T}}{\partial q}\\  &  & -\,\frac{7}{8}{(\frac{\partial {{\rm{\Gamma }}}_{T}}{\partial q})}^{2}+\frac{3}{2}\frac{{\partial }^{2}{\omega }_{T}}{\partial {q}^{2}}-\frac{\sqrt{3}}{2}\frac{{\partial }^{2}{{\rm{\Gamma }}}_{T}}{\partial {q}^{2}},\end{array}$$

where q_*_ is the wavenumber at which ${{\rm{\Gamma }}}_{T}$/${\omega }_{T}=\sqrt{3}$ and * means the values at $q={q}_{\ast }$. However, *a posteriori* analysis of the excitation spectra in fluids justifies that it is sufficient to use the first-order terms in the expansions for ${\omega }_{T}$ and ${{\rm{\Gamma }}}_{T}$, since the second *q* derivatives are small at $q={q}_{\ast }$ and can be neglected.

**Figure 2:**
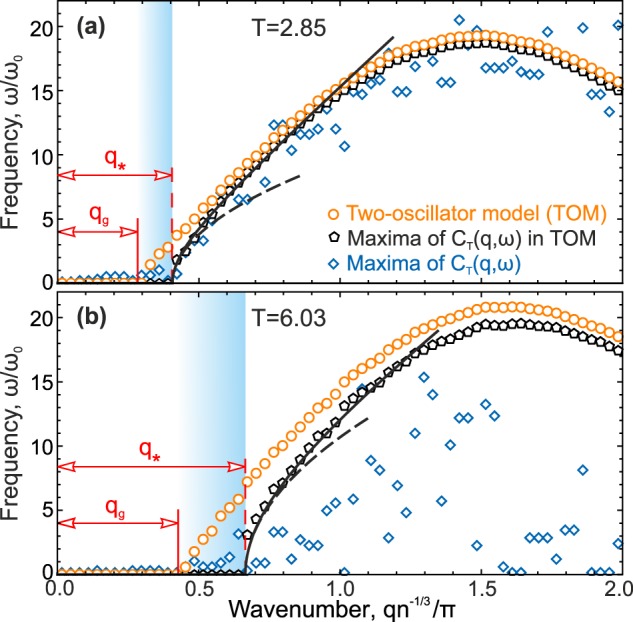
Dispersion relations in the 3D Lennard-Jones fluid at the temperature (**a**) 2.85 and (**b**) 6.03. Orange circles correspond to ${\omega }_{T}$ obtained with the two-oscillator model (5), black pentagons are maxima of the transverse part of current spectra in the two-oscillator model. Blue rhombs represent maxima of transverse current spectra ${C}_{T}(q,\omega )$ at different *q* values. The wavelength range highlighted by the gradient blue area corresponds to the transition from overdamped to non-overdamped excitations. The black solid and dashed lines are theoretical expansions Eq. (7) and its version with ${\beta }_{\ast }\equiv 0$, respectively, with the coefficients taken from the two-oscillator model.

Figure 2 presents the low-frequency branches of dispersion curves in the 3D Lennard-Jones fluid at the density $n=1$ and temperatures (a) 2.85 and (b) 6.03. The curves were obtained using the two-oscillator model (orange circles), maxima of the transverse part of current spectra ${C}_{T}(q,\omega )$ in the two-oscillator model (black pentagons), and maxima of transverse current spectra ${C}_{T}(q,\omega )$ (blue rhombs). The black solid and dashed lines correspond to Eq. (7) and its linearized variant with ${\beta }_{\ast }\equiv 0$, respectively, while the frequencies, damping rates, and *q*-derivatives were provided by the two-oscillator model (5).

Figure 2 demonstrates the following three domains in the *q*-space: (i) *q*-gap ($q < {q}_{g}$) with ${\omega }_{T}=0$, i.e., the range of missing oscillating excitations, and domains of (ii) *overdamped excitations* (${q}_{g} < q < {q}_{\ast }$) with ${\omega }_{m}=0$ at ${\omega }_{T}\ne 0$ and (iii) *non-overdamped* excitations ($q > {q}_{\ast }$) with ${\omega }_{m}\ne 0$ and ${\omega }_{T}\ne 0$. The (transition) region of overdamped oscillating excitations is shown in Fig. 2 by the gradient blue area.

At low temperatures, ${\omega }_{T}(q)$ obtained using maxima of the ${C}_{T}(q,\omega )$ part of the two-oscillator model is close to that obtained from the analysis of transverse current spectra, as shown in Fig. 2(a). At high temperatures, the two-oscillator model still yields dispersion curves ${\omega }_{T}(q)$ with a clear *q*-gap, while the method based on ${C}_{T}(q,\omega )$ maxima provides irregular points and becomes inappropriate. The observed irregular behavior is related to the flat form of ${C}_{T}(q,\omega )$ at large ${{\rm{\Gamma }}}_{T}/{\omega }_{T}$ values, at which even weak data noise (due to thermal fluctuations and limited statistics) can strongly affect a position of the detected maximum of ${C}_{T}(q,\omega )$ (e.g., it is clearly seen in ref.^53^). Second, the high-frequency branch of excitations is significantly broaden and, therefore, also affects the positions of the ${C}_{T}(q,\omega )$ maxima due to the mode mixing.

Figure 2 proves that approaches based on maxima are unsuitable for the proper analysis of the true *q*-gap where ${\omega }_{T}=0$. Comparing results represented by orange- and black-colored symbols, one can see that the discrepancy between ${\omega }_{T}$ and ${\omega }_{m}$ is maximal near the *q*-gap and exhibits a systematic difference of about 50% between ${q}_{g}$ and q_*_. Therefore, it is crucially important to properly include damping in the analysis of excitation spectra when the frequency and damping rate are comparable with each other. To accurately calculate *q*_*g*_, using discrete values of ${\omega }_{T}(q)$ obtained within the two-oscillator model, we fitted ${\omega }_{T}(q)$ in the region near the gap boundary by the linear dependence:

8 $${\omega }_{T}(q)={c}_{T}(q-{q}_{g})\theta (q-{q}_{g}),$$

where $\theta (q)$ is the Heaviside step function and ${c}_{T}$ characterizes the velocity of the overdamped low-frequency excitations. Results of $q$-gap calculations in the Lennard-Jones, IPL12, and IPL8 fluids at different temperatures are presented and discussed below.

Concluding this section, note once again that the two-oscillator model can be used to obtain accurately the parameters of the square root relation ${\omega }_{m}(q)$ (7) near the transition between overdamped and non-overdamped domains of excitations. Namely, the square root relation has been typically discussed in the context of collective excitations in fluids (see, e.g.^27,53^ and references therein).

### Excitation spectra at high temperatures

**Figure 3:**
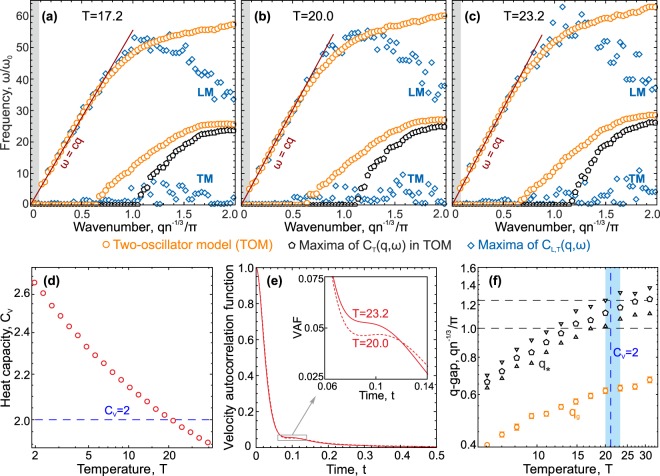
Dynamics and thermodynamics in the 3D Lennard-Jones fluid. (**a**–**c**) Dispersion relations ${\omega }_{L,T}(q)$ at temperatures $T=17.2$, 20, and 23.2, respectively, calculated using the two-oscillator model (5) (orange circles), maxima of its transverse part ${C}_{T}(q,\omega )$ (black pentagons), and maxima of the longitudinal (LM) and transverse (TM) current spectra ${C}_{L,T}(q,\omega )$ (blue rhombs). The solid red lines are theoretical asymptotic curves $\omega =cq$ given by Eq. (9). Grey zones show the regions $q < 2\pi /L$ (*L* is the size of the considered system). (**d**) Temperature dependence of the specific heat ${C}_{V}(T)$. (**e**) Velocity autocorrelation functions (VAFs) at $T=20$ and 23.2 (corresponding to the vicinity of ${C}_{V}=2$). The inset presents a zoom of the region in the grey frame to highlight the change in the VAF with an increase in the temperature. (**f**) Temperature dependencies of *q*_*g*_ and q_*_ obtained using the two-oscillator model (orange circles) and maxima of its transverse part ${C}_{T}(q,\omega )$ (black pentagons), while triangles indicate confidence intervals. The blue region corresponds to the temperature range where the VAF becomes monotonic rather than oscillatory. The vertical blue dashed line is $T\approx 21.1$ (at which ${C}_{V}=2$), while the horizontal black dashed lines are the positions of the first pseudo-Brillouin zone boundary determined by different ways.

To elaborate the results of previous sections, we studied in detail the dynamics and specific heat of the 3D Lennard-Jones fluid at the density $n=1$ in a wide range of temperatures. Using different approaches to analyze excitations spectra, we aimed to analyze changes in thermodynamics of the system, as well as collective and individual dynamics of particle motion. The results are summarized in Fig. 3.

Figure 3(a–c) present the dispersion relations of the excitation spectra at temperatures $T=17.2$, 20.0, and 23.2, respectively. The results are shown in the same manner as in Fig. 2, together with the linear asymptotic curve for the high-frequency (acoustic) branch:

9 $$\omega =cq,\,c={(\frac{\partial P}{\partial \rho })}_{T}^{1/2}{(\frac{{C}_{P}}{{C}_{V}})}^{1/2},$$

where *P* is the pressure and *C*_*V*_ and *C*_*P*_ are the specific heats at constant volume and pressure, respectively. The specific heat at constant pressure can be calculated from MD data as:

10 $${C}_{P}={C}_{V}+\frac{T}{{\rho }^{2}}{(\frac{\partial P}{\partial T})}_{P}^{2}{(\frac{\partial P}{\partial \rho })}_{T}^{-1}.$$

According to Fig. 3(a–c), Eq. (9) accurately describes the long-wavelength behavior of the high-frequency branch of excitations, typically associated with sound propagation. Calculated adiabatic indices $\gamma ={C}_{P}/{C}_{V}$ used in Eq. (9) are shown in Table 1. Trends in discrepancies between different approaches to the analysis of excitation spectra are the same as in the previous section.

**Table 1:** Adiabatic indices *γ* = *C*_*P*_/*C*_*V*_ of Lennard-Jones fluid at different temperatures.

	3D			2D		
*T*	17.2	20.0	23.2	9	12	15
*γ*	1.43	1.44	1.44	1.50	1.51	1.52

The results in Fig. 3(a–c) are shown for temperatures chosen to discuss crossover from liquid- to gas-like dynamics of fluids with increasing temperature (which is referred to as “Frenkel line^8^”). There is a set of features used in different previous works and attributed to the transition from the liquid- to gas-like state of fluids^8^: (i) *individual dynamics feature*: the velocity autocorrelation function (VAF) becomes monotonically decreasing rather than oscillatory, (ii) *collective dynamics features*: positive sound dispersion vanishes, transverse excitations extend beyond the first Brillouin pseudo-zone in the fluid, and (iii) *thermodynamic feature*: due to the absence of transverse excitations, the specific heat per particle at constant volume (in the quasi-harmonic approximation) becomes ${C}_{V}=(D+1)$/2. To discuss relations between these features, we presented in Fig. 3(d–f) results for the specific heat, velocity autocorrelation functions, and calculated *q*-gaps at different temperatures, respectively. Note that, since an unambiguous definition of the first Brillouin pseudo-zone in fluids is absent, its boundary can be estimated in the range $1 < q{n}^{-1/3}/\pi  < {(6/\pi )}^{1/3}$ in the 3D case shown in Fig. 3(f) by dashed black lines.

Analysis of Fig. 3(a–c) shows that ${q}_{\ast }{n}^{-1/3}/\pi \simeq 1$ at $T=20$, meaning that *all non-overdamped* excitations (${{\rm{\Gamma }}}_{T}/{\omega }_{T} < \sqrt{3}$ or, equivalently, ${\omega }_{T}\ne 0$ and ${\omega }_{m}\ne 0$) extend beyond the first Brillouin pseudo-zone with a farther increase in the temperature. Positive sound dispersion vanishes at the same temperatures, since orange symbols at $T=17.2$ lie slightly above the red line (9), while this is not the case at $T\ge 20.0$. Moreover, these results agree with the analysis of velocity autocorrelation functions shown in Fig. 3(e).

However, the situation with all excitations, including overdamped ones, is different, since Fig. 3(a–c) exhibit the regions of wavenumbers corresponding to overdamped excitations (${{\rm{\Gamma }}}_{T}/{\omega }_{T} > \sqrt{3}$ or, equivalently, ${\omega }_{T}\ne 0$ and ${\omega }_{m}=0$) which still exist at $T > 20$. To explain this statement, we showed in Fig. 3(f) the temperature dependencies of q_*_ (black pentagons) and *q*_*g*_ (orange circles). Here, the vertical dashed blue line corresponds to the temperature at which ${C}_{V}=2$, while the blue region marks the temperature range where the form of velocity autocorrelation function becomes monotonic instead of oscillating. The main conclusion here is that different features attributed to the Frenkel line agree with each other in general, but the collective dynamic features should be *reformulated* only for *non-overdamped excitations*, since stable transverse excitations from the first-pseudo zone do not necessarily vanish.

We found that the discussed trends are fairly generic and qualitatively the same results, as in Fig. 3, are observed for fluids with other interactions between particles. In particular, we considered fluids with inverse-power-law (IPL) repulsions ∝1/*r*^12^ (IPL12) and ∝1/*r*^8^ (IPL8). The IPL12 fluid is a Lennard-Jones fluid without the attractive branch ∝1/*r*^6^, which makes it possible to understand the role of the attractive branch of the potential in excitations of fluids. Then, comparison of results for the IPL12 and IPL8 fluids allows us to analyze trends when repulsive interactions become softer.

**Figure 4:**
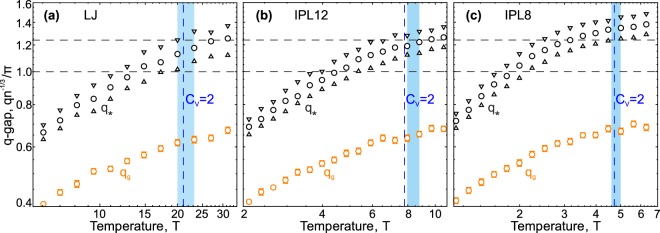
Temperature evolution of the *q*-gap in the following 3D fluids. (**a**) Lennard-Jones, (**b**) IPL12, and (**c**) IPL8 fluids at the density $n=1$. Details of the description are the same as in Fig. 3(f).

Figure 4 shows our results for the (a) Lennard-Jones, (b) IPL12, and (c) IPL8 fluids, presented in the same manner as in Fig. 3(f) (details of the description are the same). Figure 4(a) coincides with 3(f) and is reproduced here to highlight the trends appearing under the variation of the interaction. The comparison of Fig. 4(a,b) indicates that the absence of the attractive branch of interactions leads to discrepancy between thermodynamic and individual dynamic features at the Frenkel line (${C}_{V}=2$ and change in the form of the velocity autocorrelation function). When the repulsion becomes softer, agreement is recovered, but all low-frequency excitations leave the first Brillouin pseudo-zone much before the velocity autocorrelation function becomes monotonic, as seen in Fig. 4(b,c). It is remarkable that the form of temperature dependencies of the *q*-gap, ${q}_{g}(T)$, obtained from the analysis of excitations within the two-oscillator model, are weakly sensitive to the kind of interaction. Correspondence between different approaches to the Frenkel line identification requires additional detailed study.

### Excitations in 2D fluids: Enhanced anharmonicity at work

**Figure 5:**
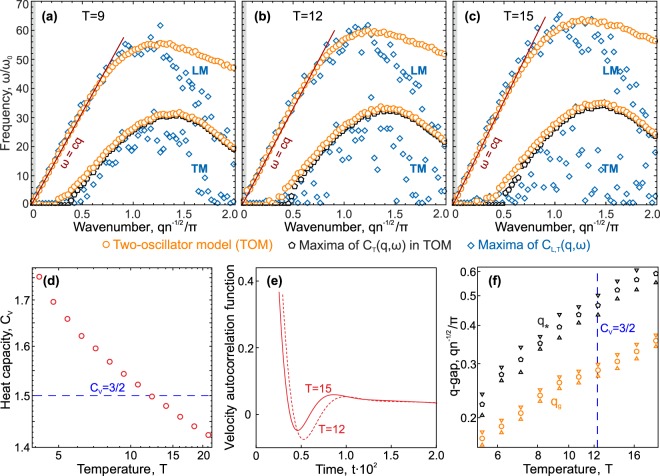
Dynamics and thermodynamics of the 2D Lennard-Jones fluid. (**a**–**c**) Dispersion relations ${\omega }_{L,T}(q)$ at temperatures $T=9$, 12, and 15, respectively, calculated using the two-oscillator model (orange circles), maxima of its transverse part ${C}_{T}(q,\omega )$ (black pentagons), and maxima of the longitudinal (LM) and transverse (TM) current spectra ${C}_{L,T}(q,\omega )$ (blue rhombs). (**d**) Temperature dependence of the specific heat ${C}_{V}(T)$. (**e**) Velocity autocorrelation functions (VAFs) at $T=12$ and 15 corresponding to ${C}_{V}\le 3/2$ in panel (d). (**f**) Temperature dependencies of *q*_*g*_ and q_*_ calculated using the two-oscillator model (orange circles) and maxima of its transverse part ${C}_{T}(q,\omega )$ (black pentagons), respectively.

Discrepancy between different dynamic and thermodynamic features attributed to the Frenkel line increases dramatically in 2D systems due to the enhanced role of anharmonicity. To illustrate this, we simulated the 2D Lennard-Jones fluid in a wide range of temperatures (see details in Methods). Then, the velocity currents, excitation spectra, specific heat, and velocity autocorrelation functions at different temperatures were analyzed in the same manner as in the case of the 3D Lennard-Jones system. The results color-coded in the same manner as in Fig. 3, are shown in Fig. 5.

We focused on the temperature region around ${C}_{V}=3/2$ (${C}_{V}=(D+1)/2$ with $D=2$), which is observed at $T\simeq 12.15$, as seen in Fig. 5(d). However, the excitation spectra shown in Fig. 5(a–c) clearly prove that a lot of non-overdamped excitations still exist in the first pseudo-zone at $T=12$, while positive sound dispersion indeed disappears near the temperature corresponding to ${C}_{V}=3/2$. The same conclusions are confirmed by the temperature dependencies of q_*_ and *q*_*g*_ shown in Fig. 5(f). Figure 5(e) justifies an oscillatory behavior of the velocity autocorrelation functions at $T=12$ and 15, while it becomes monotonic only at $T\simeq 40$ (not shown here). Thus, in contrast to the 3D Lennard-Jones system, where thermodynamic, collective, and individual dynamic features of Frenkel crossover agree with each other, the situation in 2D fluids is *essentially different*.

This discrepancy has clear physical reasons related to anharmonicity and can be explained as follows. Fluctuations in 2D systems play a much more significant role than in 3D ones. In particular, these fluctuations lead to the independent behavior of translational and orientation order parameters and rich scenarios of melting^79^. Enhanced fluctuations enlarge the amplitudes of particle motions in 2D systems, increasing the anharmonicity of direct interactions between the nearest particles. For this reason, the potential part of the interaction energy (per degree of freedom) becomes significantly smaller than 1/2. As a result, the thermodynamic feature ${C}_{V}=3/2$, which was derived in the quasi-harmonic approximation for the analysis of 3D systems^8^, becomes unsuitable for 2D fluids.

In some sense, 2D fluids of softly interacting particles behave similarly to 3D fluids of hard-sphere particles, where they move mainly beyond the interparticle interaction potential. Developed anharmonicity leads to a rapid decrease in the specific heat with increasing temperature, which is similar to that observed in strongly-anharmonic crystals^7^. However, the analysis of the behavior of the *q*-gap in 2D systems shows that it exhibits an opposite trend, which is similar to 3D fluids with soft interactions between particles. The comparison of different features associated with the Frenkel line is beyond the scope of this work and should be considered in future.

### Excitations at large *q* values: Getting back to individual dynamics

**Figure 6:**
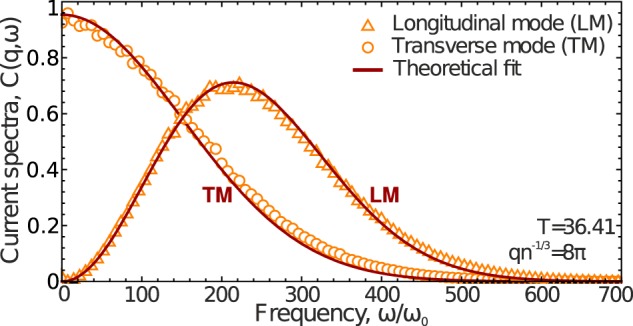
Current spectra in 3D Lennard-Jones fluids in the regime of individual dynamics of particles: at the temperature $T=36.41$ and wavenumber $q{n}^{-1/3}/\pi =8$. Symbols are MD results and the solid red lines are theoretical profiles (11) and (12) for longitudinal (LM) and transverse (TM) modes, respectively. The spectra are normalized to the maximum of transverse component.

To complement our results previously obtained for long-wavelength collective fluctuations in 3D Lennard-Jones fluids shown in Figs. 1 and 3, we considered velocity currents in a regime associated with individual particle dynamics, i.e., at high *q* values^65^. To illustrate change in velocity currents at the transition from collective to individual dynamics, we considered excitations at the temperature $T=36.41$ and density $n=1$. Figure 6 presents the sections of longitudinal (LM) and transverse (TM) modes of velocity currents ${C}_{L,T}(q,\omega )$ at $q{n}^{-1/3}/\pi =8$.

The dispersion relation for a free particle is $\omega =vq$, where *v* is the particle velocity. In thermodynamic equilibrium, the velocity distribution has a Maxwellian form and, hence, for the velocity squared (corresponding to free particle dynamics), we have

11 $${C}_{L}(q,\omega )\propto {(\frac{\omega }{q})}^{2}\,\exp \,(\,-\frac{m{\omega }^{2}}{2T{q}^{2}}),$$

where *m* is the particle mass. Note that the maximum of the distribution (11) at each fixed *q* value is reached at $\omega ={v}_{T}q$, where ${v}_{T}=\sqrt{2T/m}$ is the most probable velocity^26,58^.

Transverse current spectra in the regime of individual particle dynamics correspond to particles that can be treated as independent oscillators. As a result,

12 $${C}_{T}(q,\omega )\propto \exp \,(\,-\frac{m{\omega }^{2}}{2T{q}^{2}}).$$

Figure 6 presents the MD results for ${C}_{L,T}(q,\omega )$ (orange triangles and circles show longitudinal (LM) and transverse (TM) modes, respectively) and theoretical profiles (11) and (12). Remarkable agreement is seen between MD and theoretical results for longitudinal and transverse velocity current spectra, justifying our assumption about the individual regime of particle motion in the high-*q* limit.

The comparison of Figs 1 and 6 indicates that an increase in *q* is accompanied by the transition from collective to individual dynamics whose fingerprints are clearly seen in the form of velocity current spectra. At high *q* values, the Lorentzian profiles (4) and (5) become inaccurate and profiles (11) and (12) should be used instead of them.

## Conclusions

In conclusion, we have compared different methods for calculating excitation spectra in fluids, including the analysis of maxima of current spectra, its longitudinal and transverse components, and the joint mode analysis using the two-oscillator model to fit the total velocity current spectra. Performing MD simulations, we have studied the excitations at low- and high-temperatures in fluids of particles interacting with each other via the Lennard-Jones, IPL12, and IPL8 potentials, to understand trends arising due to potential softness and in the presence of attraction. Then, we have analyzed excitations at high temperatures corresponding to crossover from liquid- to gas-like dynamics in fluids (Frenkel line) and have studied the form of velocity current spectra in the regime of individual dynamics corresponding to excitations with high *q*-values.

We have found that the method based on the two-oscillator model provides the most accurate and comprehensive results for the frequencies and damping rates of both high- and low-frequency branches of the excitation spectra. Other considered approaches, including those based on the analysis of the transverse part of the total velocity current spectrum and on the analysis of maxima of the transverse current spectrum, can be used only for *rough estimation* of dispersion relations at low temperatures (not far from the melting line) and become unsuitable at higher temperatures.

The comparison of approaches based on the two-oscillator model and maxima of its transverse part has allowed us to distinguish three regions in the *q*-space corresponding to *q*-gap, as well as ranges of overdamped and non-overdamped low-frequency excitations. We have demonstrated that the results based on the analysis of the maxima of transverse current spectra can be obtained with the parameters of spectra calculated using the two-oscillator model. We have derived corresponding expansions near the transition between overdamped and non-overdamped regimes of low-frequency excitations.

We have analyzed the excitation spectra, specific heat, velocity autocorrelation functions, and temperature evolution of the *q*-gap in the 3D Lennard-Jones fluid at high temperatures and considered different features attributed to crossover from the liquid- to gas-like state of the fluid (Frenkel line). Considering this crossover, we observed that only non-overdamped low-frequency excitations rather than all of them leave the first pseudo-zone. However, the dynamic and thermodynamic features of the Frenkel crossover agree with each other in terms of low-frequency non-overdamped excitations instead of excitations in a broad sense (which include overdamped ones). In the case of 2D fluids, the dynamic and thermodynamic features strongly disagree with each other in general due to the increased role of anharmonicity.

We have found that the method based on the two-oscillator model is suitable for analysis of excitations in the regime of collective dynamics in 3D and 2D fluids with different interaction potentials. However, in the limit of fluctuations with high *q* values, we observed the return to the regime of individual particle dynamics, at which longitudinal and transverse current spectra are determined by Maxwellian distributions of particle velocities.

In addition to MD simulations, a powerful approach to experimental studies of fluid dynamics is provided by complex (dusty) plasmas, i.e., microparticles in a plasma discharge that acquire a negative charge and exhibit weakly damped individual particle dynamics, which can be imaged using video recording^80,81^. Particle-resolved experiments with complex (dusty) plasmas make it possible to analyze collective dynamics in strongly coupled many-body systems and, in particular, in a fluid state^82–86^. Although we considered only simple fluids in this work, we expect that the results can be applied to analyze excitations in fluid complex (dusty) plasmas.

The results of this work pave the way for detailed analysis of excitations in fluids of different natures, from simple fluids and noble gas fluids to liquid metals, molecular and complex fluids, complex (dusty) plasmas and related systems. We believe that our results will be of interest in context of various problems in condensed matter physics, chemical physics, physical chemistry, plasma physics, materials science, and soft matter.

## Methods: Details of MD Simulations

We considered systems of particles interacting via the Lennard-Jones (LJ) and inverse power law (IPLk) pair potentials^1^:

13 $${\phi }_{{\rm{L}}{\rm{J}}}(r)=4\varepsilon ({(\frac{\sigma }{r})}^{12}-{(\frac{\sigma }{r})}^{6}),\,{\phi }_{{\rm{I}}{\rm{P}}{\rm{L}}{\rm{k}}}(r)=\varepsilon {(\frac{\sigma }{r})}^{k},$$

where *r* is the distance between a pair of particles; $\varepsilon$ and *σ* are the energy and length scale of the interaction, respectively; and *k* is the exponent.

We have studied 3D and 2D fluids consisting of $N={10}^{4}$ particles in an *NVT* ensemble with a Nose–Hoover thermostat. The cutoff radius of interaction was chosen as ${r}_{c}=7.5{n}^{-1/D}$, where $n=N/V$ is the numerical density and *D* is the space dimension. The numerical time step was chosen as ${\rm{\Delta }}t=5\times {10}^{-3}\sqrt{{T}_{0}m{\sigma }^{2}/(T\varepsilon )}$ and ${T}_{0}/\varepsilon =0.01$. All simulations were performed in a cube (a square in 2D case) with a size $L=21.5$ ($L=100$ in 2D case) with periodic boundary conditions for $2\times {10}^{6}$ time steps, where the first $5\times {10}^{5}$ steps were used to equilibrate the system and the rest, to calculate the properties. Simulations were performed within the LAMMPS^87^ and HOOMD-blue^88,89^ packages.

In liquids, due to *the isotropy*, *absence of translational order*, and *strong damping of excitations* (especially with short wavelengths), a small part of the fluid inside the total volume (simulation box) can be considered as an independent subsystem. Due to this, the spectra can be calculated with fine q-resolution and in different directions (providing statistically the same results). We used this for spatial averaging of the spectra and for obtaining fine q-resolution at short wavelengths, while, in the long-wavelength region, the *q*-resolution was determined by 2*π*/*L*.
